# A new species of *Phrynoglossus* Peters, 1867; Dicroglossidae) from southeastern Bangladesh, with comments on the genera *Occidozyga* and *Phrynoglossus*

**DOI:** 10.7717/peerj.11998

**Published:** 2021-08-19

**Authors:** Scott Trageser, Hassan Al-Razi, Marjan Maria, Fahimuzzaman Nobel, Md. Asaduzzaman, Shahriar Caesar Rahman

**Affiliations:** 1 Creative Conservation Alliance, Dhaka, Dhaka District, Bangladesh; 2 Department of Zoology, Jagannath University, Dhaka, Dhaka District, Bangladesh

**Keywords:** Amphibia, Systematics, Phylogeny, Puddle frog, Conservation, Species complex, Bioacoustic, Morphology, Anura

## Abstract

We describe a new cryptic species of *Phrynoglossus* from Chattogram Division, Bangladesh based on an integrative taxonomic analysis based on morphology, phylogenetics, and bioacoustics which unambiguously support the placement of the species in the genus *Phrynoglossus*. We also present a compilation of published morphological characters for all twelve *Phrynoglossus* species and two species of *Occidozyga* as well as comments on taxonomy, morphology, and geographic distribution for the two genera. The new species is found to be most morphologically similar to *P. martensii*, however a provided set of character states visibly differentiates these two species. Finally, habitat for *Phrynoglossus swanbornorum* sp. nov. is highly fragmented and faces imminent threats from development and agriculture, and although it is confirmed to occur within government protected areas in the southeastern region of Bangladesh, few wildlife regulations are enforced within them. Thus, following IUCN criteria, we consider the new species as Endangered based on criteria B1ab(i,ii,iii,iv) + 2ab(i,ii,iii,iv).

## Introduction

*Phrynoglossus* (Peters, 1867) is a widely distributed genus in South and Southeast Asia whose occurrence is reported to reach as far west as West Bengal (India; see discussion), east to southern Jiangxi and eastern Fujian (China), and southeast to Java, Bali, Flores, Sulawesi, and the Philippines (Frost, 2021; Günther, 1864). They are semi-aquatic frogs with relatively small and stocky bodies, and are often called Javan, puddle, seep, or floating frogs due to their preference for lentic habitats; though they are not entirely restricted to them. Previous diagnoses of *Phrynoglossus* have characterized the genus as having the following combination of traits: small size, stocky habitus, short hind limbs, an indistinct or moderately distinct tympanum, no vomerine teeth, a fleshy tongue that is pointed or rounded, a flattened snout, horizontal pupils, dorsum with scattered tubercles, skin covered by an extensive mucosome, throat lining uniformly grey, tips of fingers and toes slightly swollen, length of finger I equal to II, enlarged and rounded toe tips lacking grooves, a semiaquatic life style, and inguinal amplexus (see discussion; Taylor, 1962; Iskandar, 1998; Sailo et al., 2009; Inger et al., 2017; Poyarkov et al., 2020; Köhler et al., 2021. In contrast, *Occidozyga* (*Occidozyga* Kuhl & Van Hasselt, 1822) has recently been characterized as being distinct from *Phrynoglossus* by exhibiting a slender worm-like tongue, pointed tips of fingers and toes, concealed tympanum, dryer skin not covered by extensive mucous, whitish throat lining with a longitudinal brown stripe, a fully aquatic lifestyle, and axillary amplexus (see discussion; Köhler et al., 2021).

The generic assignments of *Phrynoglossus* and *Occidozyga* have had a polyonymous history (Frost, 2021), and are currently recognized by some as monophyletic sister-taxa (Köhler et al., 2021). However, phylogenetic relationships within the clade are far from being resolved (Dubois, Ohler & Biju, 2001; Frost et al., 2006; Sailo et al., 2009; Iskandar, Arifin & Rachmansah, 2011; Inger et al., 2017; Bogisich, 2019; Chan et al., 2021; Köhler et al., 2021). *Phrynoglossus* currently comprises 12 species (Frost, 2021), with *P*. *martensii* (Peters, 1867) representing the type species and *Occidozyga* comprises two species with *Occidozyga lima* (Gravenhorst, 1829) representing the type species. A recent study resulted in the synonymy of *P. laevis vittata* (Andersson, 1942) with *P. martensii* (Poyarkov et al., 2020), and two unpublished theses have highlighted the presence of undescribed cryptic lineages within the *O. lima* complex (Chan, 2013) and the *P. martensii* complex (Bogisich, 2019). Nonetheless, only one new species of either *Phrynoglossus* and *Occidozyga* has been described in the past 60 years (*i.e.,* Iskandar, Arifin & Rachmansah, 2011), until 2021 when two additional species were described (Köhler et al., 2021; Matsui et al., 2021).

Herein, we describe a new species of *Phrynoglossus* from the lowland, semi-evergreen forests of southeast Bangladesh—a region with dense human populations. Patches of mature forest habitat remain throughout its known range, though these are highly fragmented and degraded as a result of unsustainable agricultural practices and ongoing deforestation (Gain, 2002; Kabir & Muzaffar, 2002). The new species is both morphologically and genetically distinct from its congeners, despite substantial morphological overlap with *P. martensii*. Lastly, we provide a compilation of published morphological characters for all 14 *Phrynoglossus* and *Occidozyga* species with comments on taxonomy, morphology, and geographic distribution for the genera.

## Materials & Methods

### Ethics statement

Specimen collection protocols and animal use were approved by the Department of Zoology of Jagannath University. Field work was conducted under permit 22.01.0000.101.23.2019.1922., issued by the Bangladesh Forest Department. The study was carried out in accordance with the guidelines for use of live amphibians and reptiles in field and lab research (Beaupre et al., 2004), compiled by the American Society of Ichthyologists and Herpetologists (ASIH) Herpetologists’ League HL, and the Society for the Study of Amphibians and Reptiles (SSAR).

### Taxonomy and species concept

Puddle frog generic placement follows the taxonomy proposed by Köhler et al. (2021). For recognizing species, we adhere to the General Species Concept (De Queiroz, 2005; De Queiroz, 2007). Under this concept, the only necessary property for an entity to be a recognized as a species is that it corresponds to a temporal segment of a metapopulation lineage evolving separately from other lineages. Independent evolution generates diagnostic traits detectable in a species’ morphology, vocalizations, behavior, and genetics.

### Study areas

**Figure 1:**
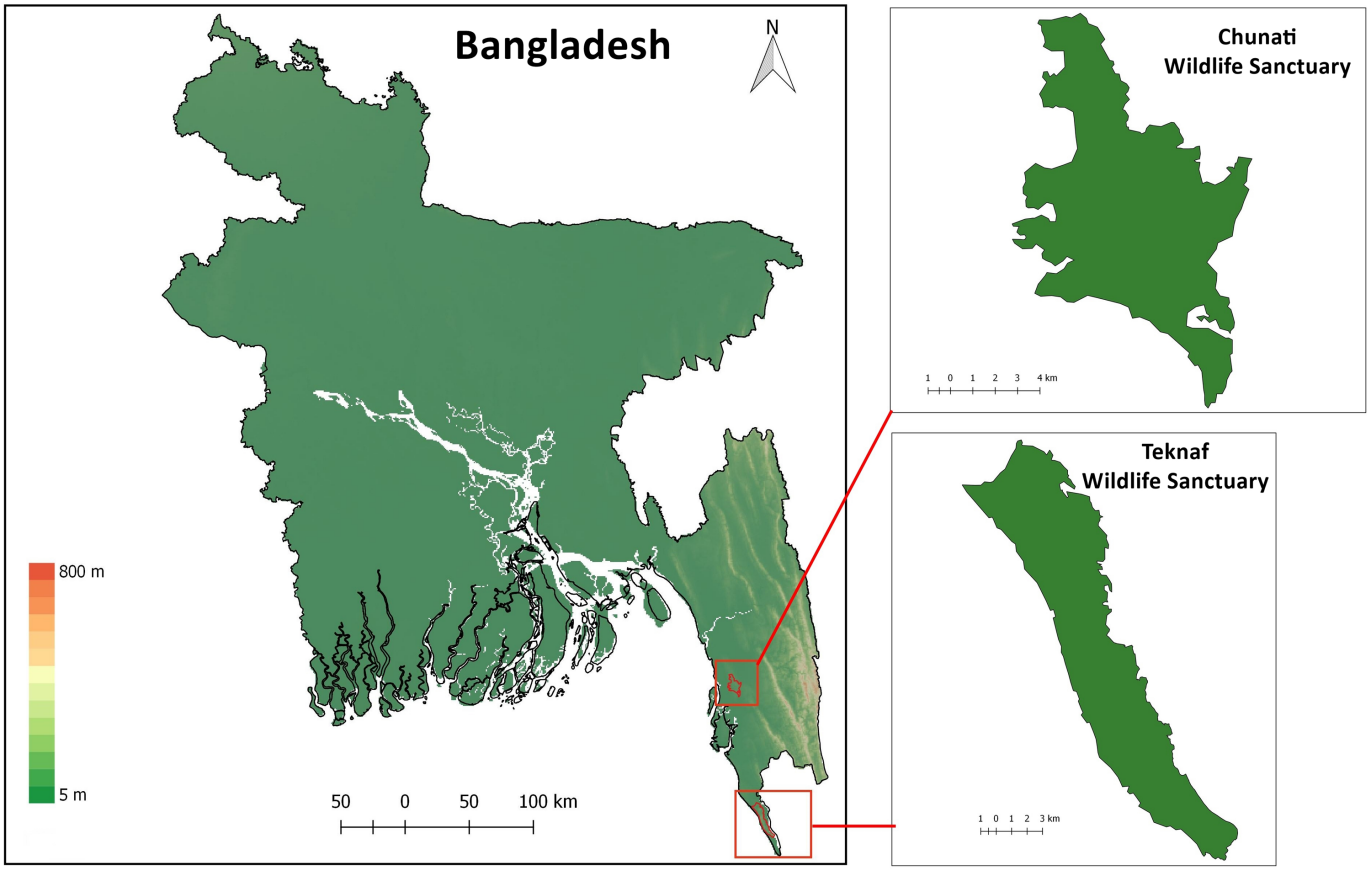
Map of Bangladesh with insets of study areas Chunati Wildlife Sanctuary and Teknaf Wildlife Sanctuary.

Chunati Wildlife Sanctuary (CWS) is situated in Chattogram District of Chattogram Division (formerly known as Chittagong Division) in southeast Bangladesh (21.900000 N 92.133333 E; Fig. 1). It was established as a Wildlife Sanctuary in 1986 with an area of 7,764 ha and consists of semi-evergreen forest 30 to 90 m above sea level, with narrow valleys and streamlets caused by broken hillocks orientated in a north-south direction. The area experiences a moist, subtropical climate with a low range of temperature and humidity variation with November to February being the coldest and driest months. The sanctuary has experienced substantial degradation in recent years due to illicit felling of trees, cultivation of betel-leaf, and creation of rice paddies as part of its management scheme. As a result, the sanctuary has lost most of its natural forest (Gain, 2002; Kabir & Muzaffar, 2002). Forest coverage in 2015 was reduced to 60% (Rahman et al., 2016). Trees such as *Dipterocarpus* sp., *Artocarpus* sp., *Albizia* sp., *Quercus* sp., *Syzygium* sp., and *Lagerstroemia* sp. still occur in CWS although they are mostly found as isolated individuals or in small, fragmented patches.

Teknaf Wildlife Sanctuary (TWS) is situated in Cox’s Bazar District of Chattogram Division, near Myanmar (20.887020 N, 92.298954 E; Fig. 1; Rosario, 1997). It was initially granted formal protection in 1983 as a Game Reserve and its total area is approximately 11,651 ha (Green, 1987). While TWS once supported primary evergreen and semi-evergreen forests, these forests have now been largely replaced with human-modified landscapes and only degraded forest remains (Alam et al., 2015). The reserve is longitudinally narrow, running roughly 28 km in a north-south direction and 3–5 km east–west (Uddin et al., 2013) with linear hill tracts reaching as high as 700 m above sea level. Between 130 and 940 mm of rain usually falls in May through October which causes numerous tributaries of the Naf river to flow through TWS mainly during the monsoon season. Average temperature in TWS can range from 15 °C to 32 °C (BBS, 2011).

### Specimen collection

Four adult males and one adult female were collected along a roadside, adjacent to mature forest, near Chunati Wildlife Sanctuary (21.93029 N, 92.06316 E) on June 30, 2019. Collected specimens were euthanized using 20% benzocaine and were fixed in 95% for five hours and preserved in 70% EtOH. Muscle samples were preserved in 95% EtOH. Color of live specimens was photographed and recorded in the field during specimen collection. All specimens collected were deposited in the Shahid Rafique Special Specimen Collection (SRSSC), Department of Zoology, Jagannath University, Dhaka.

### Morphological data

Morphometric measurements were taken of the specimens with digital calipers (to the nearest 0.01 mm) after preservation. The following measurements were taken (Iskandar, Arifin & Rachmansah, 2011): snout-vent length (**SVL**): from tip of snout to vent; head length (**HL**): distance between tip of snout to the rear of the mandible; head width (**HW**): at angle of jaw; eye diameter (**ED**): horizontal diameter of the eye; tympanum diameter (**TD**): maximum diameter of the tympanum; eye-nostril distance (**EN**): distance between anterior canthus of eye and the nostril; snout length (**SL**): from anterior canthus of eye to tip of snout; nostril-snout distance (**NS**): distance from the nostril to the tip of the snout; interorbital distance (**IOD**): least distance between proximal edges of upper eyelids; internarial distance (**IND**): least distance between nostrils; upper eyelid width (**UEW**): distance of the upper eyelid measured from inner edge to outer edge; thigh length (**TL**): distance from the middle of vent to knee; shank length (**SHL**): distance between knee and heel; foot length (**FOL**): from the base of the inner metatarsal tubercle to the tip of toe IV; hand length (**HAL**): from the base of the outer palmar tubercle to the tip of finger IV; lengths of 1st to 4th fingers (**FL I to FL IV**): from the base of the palm to the tip of the respective finger; lengths of 1st to 5th toes (**TL I to TL V**): from the base of proximal subarticular tubercle to tip of the respective toe.

### DNA extraction and amplification

DNA was extracted from muscle tissue using a standard protocol following Vences et al. (2012). A small section of thigh muscle tissue was excised from five specimens for extraction, from which, mitochondrial 16S ribosomal RNA gene was amplified. The PCR amplification and sequencing of the 16S rRNA gene were done following Palumbi et al. (1991) and Bossuyt et al. (2004) respectively. Primers 5′-GCCTGTTTATCAAAAACAT-3′(16Sar-L) and 5′-CCGGTCTGAACTCAGATCACGT-3′(16Sbr-H) were used as forward and reverse primers for 16S (Palumbi et al., 1991). PCR amplifications were performed in a 20 µl reaction volume containing 10 µl Master Mix, 1 µl T DNA (concentration 25–65 ng/µl), 1 µl forward primer (concentration 10–20 pMol), 1 µl reverse primer (concentration 10–20 pMol), and 7 µl nuclease-free water. Performed cycling conditions were as follows: an initial denaturing step at 95 °C for 3 min; 40 cycles of denaturing at 95 °C for 30 s, annealing at 50 °C for 30 s, extending at 72 °C for 45 s, and a final extending step of 72 °C for 5 min. The amplified product was sequenced at 1st Base Laboratories, Malaysia.

### Phylogenetic analyses

Homologous sequences were obtained from GenBank (http://blast.ncbi.nlm.nih.gov/Blast.cgi) (Table 1) for all species of *Phrynoglossus* and *Occidozyga* with the exception of four species with no available 16S sequences in Genbank—*P. floresianus*, *P. celebensis*, *P. semipalmatus* and *P. tompotika*. *Limnonectes limborgi* (Sclater, 1892) was selected as the outgroup to align with the analysis of Köhler et al. (2021). Sequences were aligned using the MUSCLE tool in MEGA 7 (Kumar, Stecher & Tamura, 2016) and alignments were checked visually. Alignment gaps were treated as missing data. The best substitution model, General Time Reversible with proportion of invariable sites and gamma distribution (GTR+I+G), was selected using the Akaike Information Criterion (AIC) and (GTR+G) Bayesian information criteria (BIC) in jModelTest v2.1.2. Maximum likelihood phylogenetic analyses were performed using the RAxML v4.0 Geneious plugin (Stamatakis, 2006) with 1,000 bootstrap replicates. Bayesian phylogenetic inference analysis was performed in MrBayes 3.2.4 (Ronquist et al., 2012). We performed a MCMC Bayesian analysis that consisted of two simultaneous runs of 1 million generations and sampled every 100 generations. The first 25% of the sampled trees were discarded as burn-in and the remaining trees were used to create a consensus tree and to estimate Bayesian posterior probabilities (BPPs). The trees were visualized and edited in FigTree 1.4.4 (http://tree.bio.ed.ac.uk/software/figtree). Additionally, 16S pairwise genetic distances (uncorrected p) for six *Phrynoglossus* species including *Phrynoglossus* sp. nov. were calculated using MEGA 7.0 (Kumar, Stecher & Tamura, 2016).

**Table 1:** *Occidozyga and Phrynoglossus* 16S rRNA sequence information. *Phrynoglossus swanbornorum* sp. nov. sequences were generated during this study and homologous sequences were obtained from GenBank. Species name, collection location, specimen voucher number, sequence accession number, and source of data are provided.

	**Species**	**Location**	**Voucher**	**GenBank** **16S rRNA accession numbers**	**Source**
1	*P. swanbornorum* **sp. nov.**	Chattogram, Bangladesh	JnUZool-A0719	MN705433	This study
2	*P. swanbornorum* **sp. nov.**	Chattogram, Bangladesh	JnUZool-A0819	MN705434	This study
3	*P. swanbornorum* **sp. nov.**	Chattogram, Bangladesh	JnUZool-A0919	MN705435	This study
4	*P. swanbornorum* **sp. nov.**	Chattogram, Bangladesh	JnUZool-A1019	MN705436	This study
5	*P. swanbornorum* **sp. nov.**	Chattogram, Bangladesh	JnUZool-A1117	MN705437	This study
6	*P. myanhessei*	Thanlyin, Yangon, Myanmar	SMF 103797	MW217501	Köhler et al. (2021)
7	*P. myanhessei*	Thanlyin, Yangon, Myanmar	SMF 103798	MW217502	Köhler et al. (2021)
8	*P. myanhessei*	Thanlyin, Yangon, Myanmar	SMF 103800	MW217503	Köhler et al. (2021)
9	*P. myanhessei*	Yangon, Myanmar	USNM:Herp:587107	MG935920	Mulcahy et al. (2018)
10	*P. myanhessei*	Bago, Myanmar	USNM:Herp:587105	MG935916	Mulcahy et al. (2018)
11	*P. myanhessei*	Yangon, Myanmar	USNM:Herp:587395	MG935918	Mulcahy et al. (2018)
12	*P. myanhessei*	Yangon, Myanmar	USNM:Herp:587402	MG935917	Mulcahy et al. (2018)
13	*P. martensi*	Myanmar	USNM:Herp:586940	MG935942	Mulcahy et al. (2018)
14	*P. martensi*	Myanmar	USNM:Herp:586942	MG935941	Mulcahy et al. (2018)
15	*P. martensi*	Myanmar	USNM:Herp:586943	MG935940	Mulcahy et al. (2018)
16	*P. martensi*	Myanmar	USNM:Herp:586930	MG935939	Mulcahy et al. (2018)
17	*P. martensi*	Myanmar	USNM:Herp:586931	MG935938	Mulcahy et al. (2018)
18	*P. martensi*	Myanmar	USNM:Herp:586937	MG935932	Mulcahy et al. (2018)
19	*P. martensi*	Myanmar	USNM:Herp:586938	MG935931	Mulcahy et al. (2018)
20	*P. martensi*	Myanmar	USNM:Herp:586939	MG935930	Mulcahy et al. (2018)
21	*P. martensi*	Myanmar	USNM:Herp:586941	MG935929	Mulcahy et al. (2018)
22	*P.* sp.	Yangon, Myanmar	MBM-JBS19932	MG935921	Mulcahy et al. (2018)
23	*P.* sp.	Yangon, Myanmar	USNM:Herp:587389	MG935919	Mulcahy et al. (2018)
24	*P.* sp.	Yangon, Myanmar	USNM:Herp:587386	MG935914	Mulcahy et al. (2018)
25	*O. lima*	Myanmar	USNM:Herp:586925	MG935926	Mulcahy et al. (2018)
26	*O. lima*	Myanmar	USNM:Herp:586927	MG935928	Mulcahy et al. (2018)
27	*O. lima*	Myanmar	USNM:Herp:586926	MG935927	Mulcahy et al. (2018)
28	*O. lima*	Myanmar	USNM:Herp:586924	MG935925	Mulcahy et al. (2018)
29	*O. lima*	Sagaing, Myanmar	USNM:Herp:520376	MG935924	Mulcahy et al. (2018)
30	*O. lima*	Mandalay, Myanmar	MBM-JBS5405	MG935923	Mulcahy et al. (2018)
31	*O. berbezus*	Malaysia:Matang	KUHE:17327	LC593607	Matsui et al. (2021)
32	*P. magnapustulosus*	Thiland	GK_7395	MW217488	Köhler et al. (2021)
33	*P. magnapustulosus*	Thiland	GK_7396	MW217487	Köhler et al. (2021)
34	*P. magnapustulosus*	Thiland	GK_7916	MW217489	Köhler et al. (2021)
35	*P. laevis*	Philippines: Pasonanca,	KU 314470	MT820168	Chan et al. (2021)
36	*P. laevis*	Philippines: Pasonanca,	KU 319796	MT820169	Chan et al. (2021)
37	*P. sumatranus*	Selangor, Malaysia	FRIM 1132	MT820181	Chan et al. (2021)
38	*P. sumatranus*	Selangor, Malaysia	FRIM 1133	MT820182	Chan et al. (2021)
39	*P. sumatranus*	Selangor, Malaysia	FRIM 1936	MT820183	Chan et al. (2021)
40	*P. diminutivus*	Philippines: Pasonanca,	KU 321225	MT820199	Chan et al. (2021)
41	*P. diminutivus*	Philippines: Pasonanca,	KU 321226	MT820200	Chan et al. (2021)
42	*P. diminutivus*	Philippines: Pasonanca,	KU 321227	MT820201	Chan et al. (2021)
43	*P. baluensis*	Sabah, Myanmar	FMNH 242747	DQ283143	Frost et al. (2006)
44	*Limnonectes limborgi*	Myanmar	GK_7110	MW217495	Köhler et al. (2021)

### Call recording and analysis

Call analysis is based on a single recording of advertisement calls from a single male *Phrynoglossus* sp. nov. obtained by SJT on June 30, 2019 at 20:00 h during light rain and ambient air temperature measuring 30.1 °C. Advertisement call recording was made with an LG V30 smartphone with sampling rate of 44.1 kHz and 32-bit resolution. The smartphone was placed approximately 1.5 m from the calling male.

The original call recording file was converted from .m4a format to .wav format using Adobe^®^ Audition^®^ (version 13.0.1.35; Adobe Inc., San Jose, CA, USA). Adobe Audition sound removal process was used to generate a recording to facilitate measuring temporal variables. Sound removal was applied with the following settings: sound model complexity 60, sound refinement passes 150, content complexity 60, and content refinement passes 150. Sound model was trained and applied with segments from 1.5–3 s and 10–11.5 s.

Call duration was defined as the length of a note. Call period was the time interval from the beginning of one note to the beginning of the next note. Call repetition rate was the inverse of note period. The number of pulses was the number of pulses in a note. Pulse rate was calculated as the number of pulses in a note divided by call duration. Call bandwidth was measured using the “Freq 5%” and “Freq 95%” measurement functions in Raven Pro 1.61. Dominant frequency was defined as the frequency with the most energy. Spectrogram configuration was set at Hann window of 512-sample window size and 256-sample hop size with 50% frame overlap and 86.1-Hz frequency grid spacing. Each call was analyzed for both temporal and spectral domains (Raven Pro 1.6.1; Cornell Lab of Ornithology, New York, USA), following the recommendations of Köhler et al. (2017).

### Conservation

Extinction risk was evaluated based on IUCN Red List categories and Criteria (2012). Area of occupancy and extent of occurrence were calculated using the software, GeoCAT, with a default cell size of 2 km^2^.

### Nomenclature

The electronic version of this article in Portable Document Format (PDF) will represent a published work according to the International Commission on Zoological Nomenclature (ICZN), and hence the new names contained in the electronic version are effectively published under that Code from the electronic edition alone. This published work and the nomenclatural acts it contains have been registered in ZooBank, the online registration system for the ICZN. The ZooBank LSIDs (Life Science Identifiers) can be resolved, and the associated information viewed through any standard web browser by appending the LSID to the prefix http://zoobank.org/. The LSID for *Phrynoglossus swanbornorum* sp. nov. is: urn:lsid:zoobank.org:pub:DE8850C6-0A7B-4EA2-AC9E-69EE6EFBF754. The online version of this work is archived and available from the following digital repositories: PeerJ, PubMed Central and CLOCKSS.

## Results

### Phylogenetic relationships of *Phrynoglossus*.

**Figure 2:**
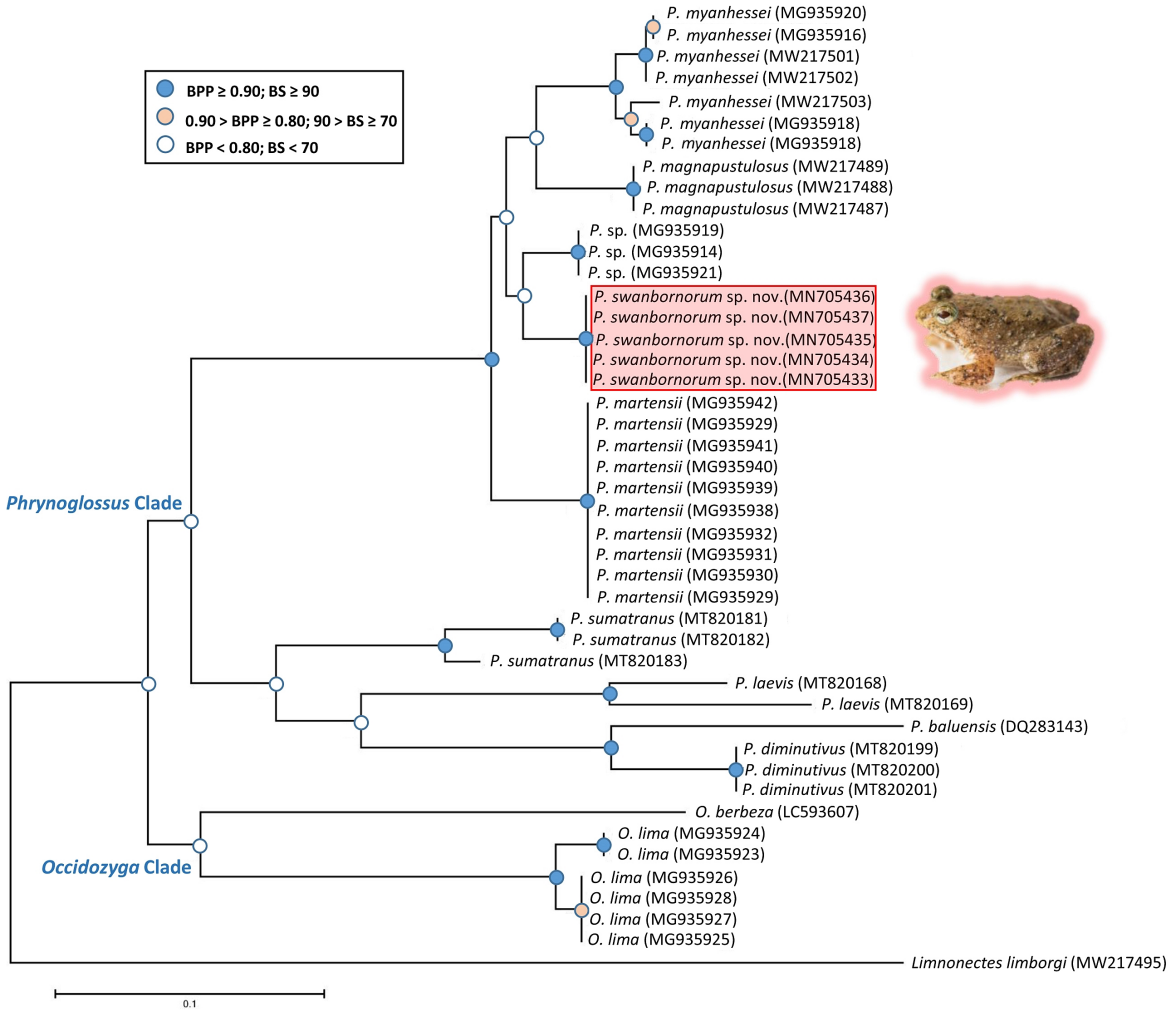
Consensus tree of merged Bayesian Inference and Maximum Likelihood analyses with Bayesian posterior probabilities and bootstrap supports.

The ML and BI analyses resulted in essentially identical topologies and were integrated in the consensus tree derived from the analyses of 2001 bp of 16S rRNA gene fragments alignment (Fig. 2), in which all intraspecies nodes were sufficiently supported with the Bayesian posterior probabilities (BPP > 0.90) and the bootstrap supports (BS > 70). Most interspecies nodes had poor support (BPP < 0.80; BS < 70) with the exception of the node supporting *P. myanhessei*, *P. magnapustulosa*, *P. swanbornorum* sp. nov., *P.* sp., and *P. martensii*. A second major *Phrynoglossus* clade comprises *P. sumatrana*, *P. laevis*, *P. baluensis*, and *P. diminutiva* and is poorly supported (BPP < 0.80; BS < 70). Support for the *Phrynoglossus* and *Occidozyga* clades is similarly poorly supported (BPP < 0.80; BS < 70). Although *P. martensii* is morphologically similar to our new species, its genetically distinctiveness is strongly supported (BPP > 0.90; BS > 90)*.*

The uncorrected p-distances for the 16S rRNA gene, which are interpreted as interspecific distances, were lowest between our new species and another undescribed species from Yangon, Myanmar (*p* = 3.9%) and *P. magnapustulosa* (*p* = 4.9%, Table 2). The highest interspecific distances were between *O. lima* and *P. laevis* (Günther, 1858) (*p* = 19.9%, Table 2). The average divergence (p-distance) within the new species ranged from 0.2% to 0.7% and average divergence between congeners ranged from 5.3% (*P. myanhessei*) to 18.3% (*O. berbeza*; Table 2). This level of divergence in the 16S rRNA gene is typically seen in many other frog species (Fouquet et al., 2007; Vences et al., 2005), thereby justifying the status of *Phrynoglossus swanbornorum* sp. nov. as a new species.

### Species description

**Table:** 

**Figure 3:**
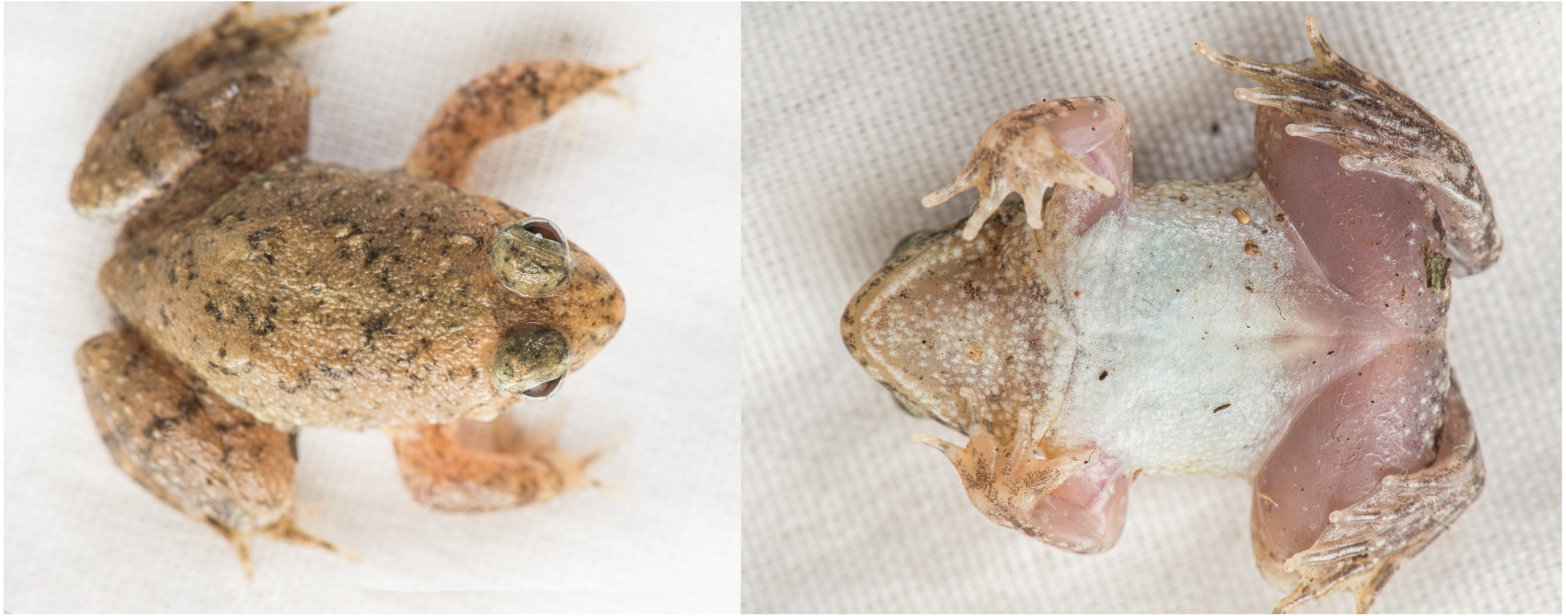
Photographs of live holotype JnUZool-A0719.

**Figure 4:**
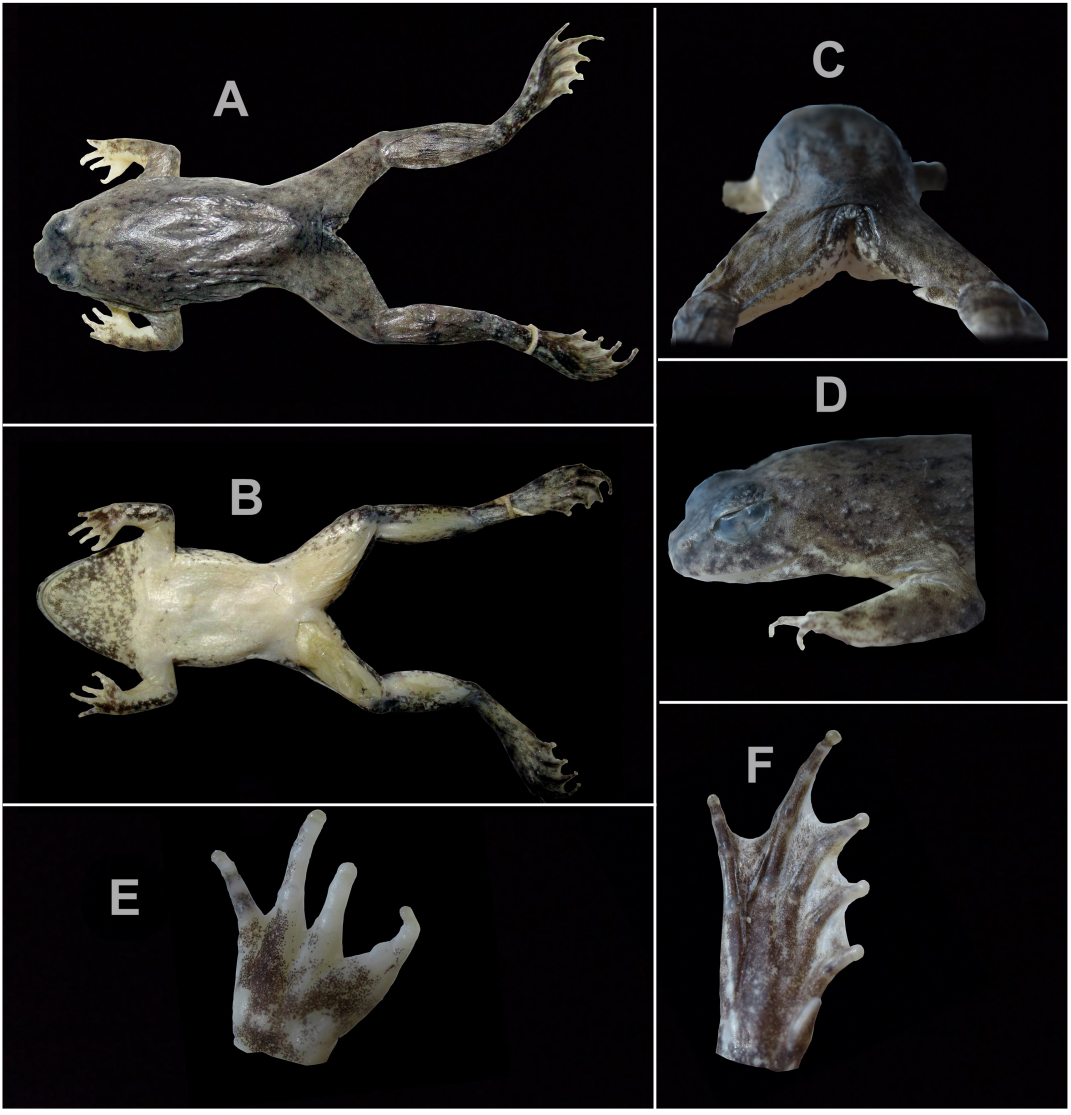
Photographs of holotype JnUZool-A0719. (A) Dorsal aspect, in preservation (B) ventral aspect, in preservation (C) posterior aspect, in preservation (D) posterior in lateral aspect, in preservation (E) hand in dorsal aspect, in preservation (F) foot in dorsal aspect, in preservation.

**Figure 5:**
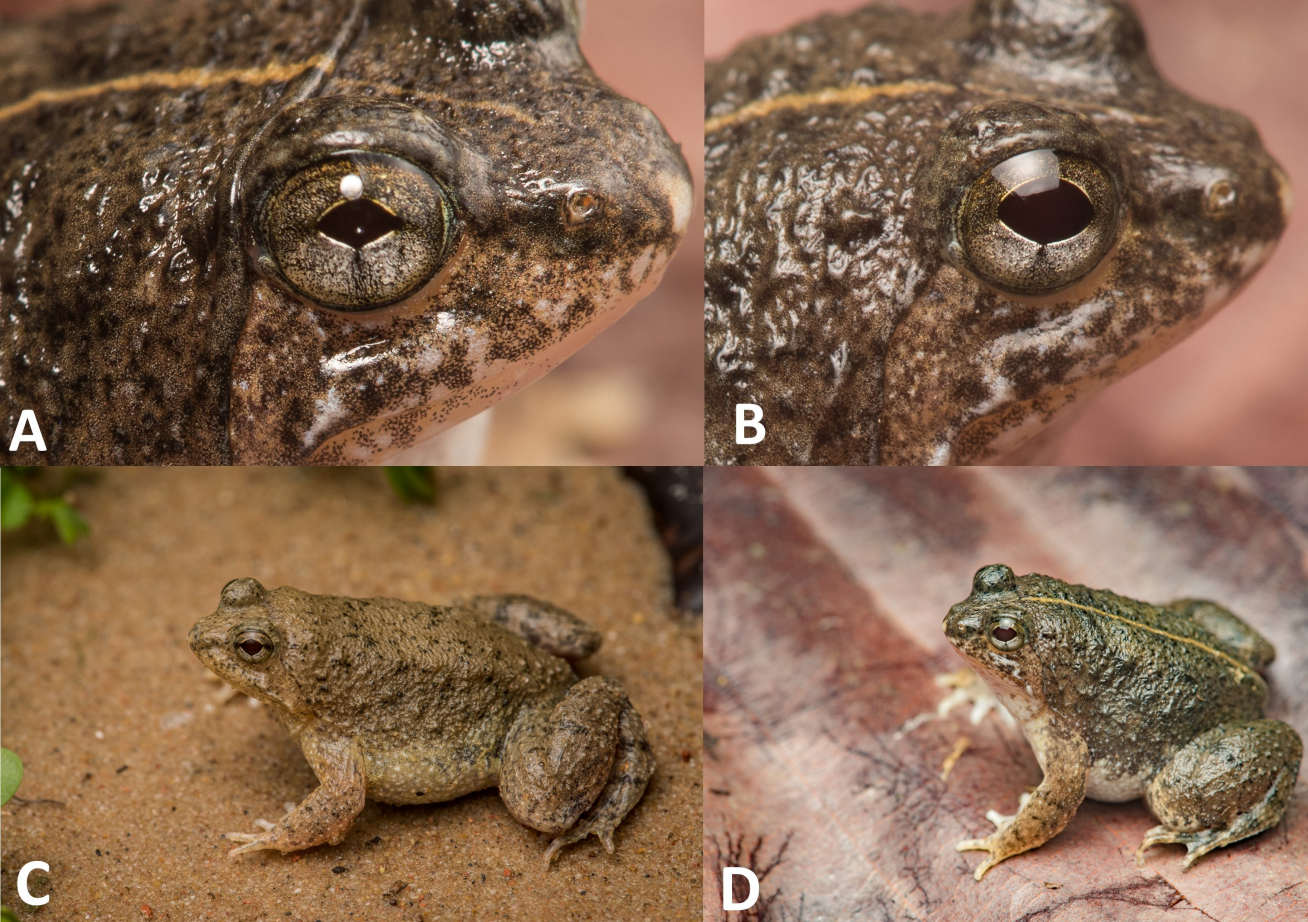
*Phrynoglossus swanbornorum* sp. nov. individuals. (A) Individual 1 with diamond shaped pupil, in life (B) individual 1 with ovoid shaped pupil, in life (C) individual 2 exhibiting no dorsolateral line, in life (D) individual 1 exhibiting dorsolateral line, in life.

**Figure 6:**
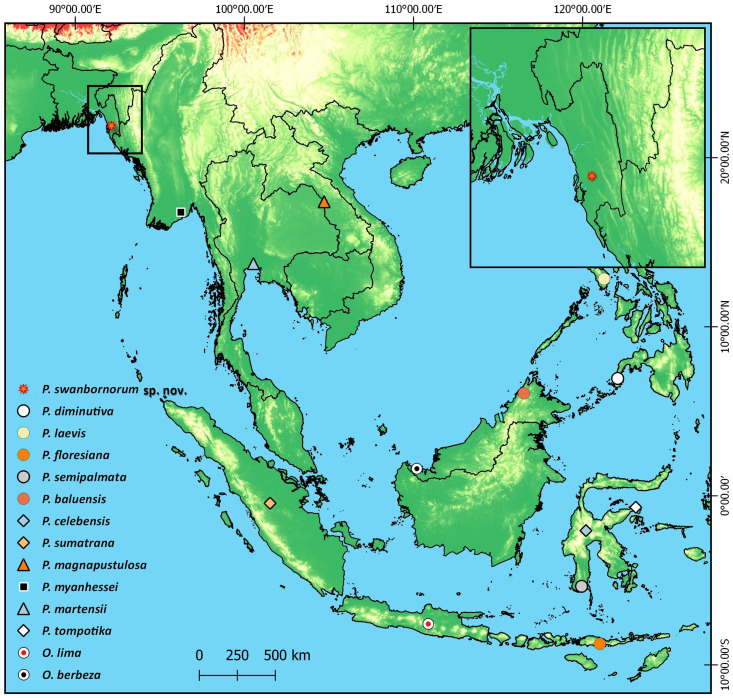
Type localities of all 14 species of *Occidozyga* and *Phrynoglossus* species.

*Phrynoglossus swanbornorum* **sp. nov.**
LSID urn:lsid:zoobank.org:pub:DE8850C6-0A7B-4EA2-AC9E-69EE6EFBF754
Figs. 3–6

**Recommended vernacular name.** English: Swanborn’s Puddle Frog. Bangla: “Chattgai ar gata bang,” which translates to English as “puddle frog from Chattogram.”

**Holotype.** (Figs. 3–5). JnUZool-A0719, adult male from a roadside ditch near Chunati Wildlife Sanctuary, Chattogram Division, Bangladesh (21.937533 N, 92.063010 E, ca. 33 m a.s.l., Fig. 1), collected on June 30, 2019 by Fahimuzzaman Nobel.

**Paratypes.** Three adult males (JnUZool-A0919, JnUZool-A1019, and JnUZool-A1119) and one adult female (JnUZool-A0819) with the same data and place as the holotype.

**Chresonymy.** Within Bangladesh, this species was first reported from southeast Bangladesh in the Teknaf peninsula (Khan, 1997; Khan, 2001), but without photographic evidence or reference to museum specimens (see Distribution).

**Generic placement.** The new species is assigned to the genus *Phrynoglossus* based on the following combination of shared adult characters: lacking vomerine teeth and exhibiting a stocky habitus, flattened snout, dorsum or flanks with scattered tubercles, short arms, nuptial pads, horizontal pupils, bony style, forked omosternum, pigmented eggs, distinct or indistinct supratympanic fold, reduced to absent metacarpal webbing, moderate to extensive metatarsal webbing, an elongated inner metatarsal tubercle, indistinct and small tympanum, feebly to moderately developed toes discs, SVL between 15 mm and 61.6 mm in length, and females are larger than males. These adult characters agree with previous descriptions of the genus (see discussion; Taylor, 1962; Iskandar, 1998; Sailo et al., 2009; Inger et al., 2017; Poyarkov et al., 2020; Köhler et al., 2021). Larval characters are unknown.

**Table 2:** Uncorrected *p*-distances for the 16s rRNA gene.

		1	2	3	4	5	6	7	8	9	10	11	12	13	14	15
1	*P. swanbornorum* sp. nov.															
2	*P. swanbornorum* sp. nov.	0.2	–													
3	*P. swanbornorum* sp. nov.	0.2	0.0	–												
4	*P. swanbornorum* sp. nov.	0.2	0.0	0.0	–											
5	*P. swanbornorum* sp. nov.	0.7	0.5	0.5	0.5	–										
6	*P. myanhessei*	5.3	5.3	5.3	5.3	5.3	–									
7	*P. martensii*	5.8	5.8	5.8	5.8	5.8	7.3	–								
8	*Phrynoglossus* sp.	3.9	3.9	3.9	3.9	3.9	3.9	6.4	–							
9	*P. magnapustulosus*	4.9	4.9	4.7	4.9	4.9	6.0	5.9	6.5	–						
10	*P. laevis*	15.4	15.4	15.4	15.4	15.4	16.8	14.8	16.2	16.1	–					
11	*P. sumatranus*	15.9	15.9	15.4	16.0	16.0	15.3	15.3	15.1	15.4	15.3	–				
12	*P. diminutivus*	18.0	18.0	17.5	17.9	17.9	19.6	19.3	18.5	18.4	17.8	16.2	–			
13	*P. baluensis*	17.5	17.5	17.1	17.4	17.4	19.1	16.7	18.7	18.1	18.7	15.8	9.6	–		
14	*O. berbeza*	17.8	17.8	18.3	18.2	18.2	19.2	192	18.2	18.9	17.6	17.6	16.6	18.8	–	
15	*O. lima*	16.2	16.2	15.8	16.2	16.2	17.9	16.9	15.7	16.4	19.9	18.0	18.9	16.8	19.3	–

**Diagnosis.** The new species presents the following characteristics: (1) relatively small sized *Phrynoglossus* (adult males = 23.15–28.56 mm SVL, adult female = 30.56 mm); (2) stocky habitus; (3) short arms relative to body size (FLL/SVL 0.19%; range 0.18−0.20%, *n* = 5); (4) head wider than long (HL/HW 0.73%; range 0.69−0.77%, *n* = 5); (5) snout slightly projecting, triangular in ventral aspect, smaller than horizontal diameter of eye, and flat in lateral aspect; (6) nostril closer to tip of snout than to eye (NS 0.48−0.51 mm, EN 0.92–109 mm); (7) brown dorsum transitioning to brownish grey on flanks with black speckling concentrated around scattered tubercles, nares, mouth, and above eyes; (8) venter uniform cream white, becoming brown with white mottling and groups of minute dark-grey flecks present in the gular region; (9) tuberculate dorsum; (10) laterally oriented eyes and nares; (11) horizontal pupils; (12) vomerine teeth absent; (13) tongue rounded without notch behind; (14) vocal sac single, median, internal, and subgular in males; (15) canthus rostralis rounded; (16) tympanum indistinct, small, and rounded; (17) supratympanic fold distinct and transverses interorbitally at the posterior edge of the eye; (18) fingertips rounded without discs; (19) nuptial pads present in males; (20) palmar tubercles present; (21) metacarpal webbing absent; (22) toe discs feebly developed; (23) inner metatarsal tubercle elongated and compressed, present at base of 1st toe; (24) fringe of skin on outer side of fifth metatarsal absent; (25) tarsal tubercle absent; (26) tarsal fold absent; (27) metatarsal webbing moderate; and (28) lateral line absent. Details of these characteristics are provided in Table 3.

**Description of holotype.** Adult male (JnUZool-A0719). SVL = 24.89 mm (Fig. 3; Table 3; all measurements in mm); head wider than long (HW/HL ratio 145.8%); snout triangular in ventral aspect, shorter than eye diameter (ED/SL ratio 69.5%), flat in lateral aspect; canthus rostralis rounded; loreal region slightly convex; interorbital region flat and smaller than the upper eyelid (IOD/UEW ratio 77.6%) and internarial distance (IOD/IND ratio 91.7%); nostrils rounded, directed laterally, and closer to tip of snout than to eye (NS/EN ratio 47.6%); tympanum small, rounded, close to eye, and covered by skin but with outline faintly visible (TD = 1.21); supratympanic fold distinct and transverses interorbitally at the posterior edge of the eye; eyes relatively large (ED = 3.02) and protruding; pupils horizontal, and ovoid when dilated and diamond when constricted; vocal sac single, median, internal, and subgular; numerous minute tubercles present on the anal portion and the base of the foot; fine dorsolateral ridges on shank.

Forelimb length approximately equal to hand length (FLL /HAL ratio 98.4%); relative lengths of fingers IV<II<I<III (FL I = 1.75; FL II = 1.56; FL III = 2.51; FL IV = 1.45); fingertips rounded and without disk; toe discs feebly developed; webbing between fingers absent; subarticular tubercles well developed and rounded; number of subarticular tubercles in fingers: *I* = 1, II = 1, III = 2, IV = 1; supernumerary tubercles indistinct; nuptial pad present.

Hind limbs long, shank longer than thigh (SHL/TL ratio 113.4%) and foot (SHL/FOL ratio 109.9%); relative toe length I<V<II<III<IV (TL I=2.10; TL II = 3.80, TL III = 5.32; TL IV = 7.23; TL V=3.63); toes with small discs and circummarginal grooves absent; webbing moderate; webbing formula (fingers: I $ \frac{11}{3} $ - 2 II $ \frac{11}{3} $ - $ \frac{21}{3} $ III $ \frac{12}{3} $ - 3 IV $ \frac{31}{3} $ - $ \frac{11}{3} $ V) (Figs. 4D, 4E); inner and outer metatarsal tubercles absent; subarticular tubercle present and rounded (toe: I=1, II = 1, III = 1, IV = 2, V=2); inner metatarsal tubercle elongated (1.86 mm), compressed, and present at base of 1st toe; fringe of skin on outer side of fifth metatarsal absent.

**Coloration of holotype in life.** In life, all specimens exhibited a brown dorsum transitioning to brownish grey on flanks with black speckling concentrated around scattered tubercles, nares, mouth, and above eyes; narrow, yellow dorsolateral stripe present or absent; forelimbs with black spots and hind limbs with black bands; dorsal tubercles on flanks tipped in brownish white to white; shanks with incomplete dark banding; venter uniform cream white; gular region brown with white mottling and scattered groups of dark-grey flecks; ventral side of feet translucent with heavy black speckling; ventral side of hands light tan with moderate black speckling; pupil ovoid when dilated and diamond when constricted; and iris has many fine, dark spots which can create indistinct reticulations which are predominantly silver and distally bordered by brown below the pupil, dark blotches in the median region, and predominantly brown with fine, dark reticulations above the pupil (Fig. 5).

**Coloration of holotype ethanol.** In ethanol, the pattern described above has not changed, although color has faded. Brownish tint has faded to dark grey on dorsum and light grey on tympanum; webbing coloration faded from pinkish to cream white; ventral side of hands faded to light grey; skin covering the tympanum appears thinner in preserved specimens which exaggerates the appearance of the tympanum; and other dehydration artifacts are present.

**Variation and sexual dimorphism.** The only preserved female (30.6 mm) is slightly larger than the largest male (28.6 mm). There are no other notable differences among the type series, however a photographed individual of *Phrynoglossus swanbornorum* sp. nov. from Teknaf Wildlife Sanctuary exhibits a distinct dorsolateral stripe and was documented constricting its ovoid pupil to appear diamond shaped (Figs. 5B and 5D). Variation in size and body proportions in this species are given in Table 3.

**Table 3:** Morphometric measurements *Phrynoglossus swanbornorum* sp. nov. Character measurements to the nearest 0.01 mm are provided for the holotype and paratypes.

		***P. martensii***	***P. magnapustulosus***	***P. myanhessei***	***P. swanbornorum***
		♂7 — ♂8	♂8 —♂12	♂9 —♂6	♂4 —♂1
SHL/SVL	males	0.441–0.509 (0.479 ± 0.021)	0.442–0.557 (0.509 ± 0.036)	0.457–0.571 (0.506 ± 0.032)	0.428–0.449 (0.440 ± 0.009)
	female	0.436–0.479 (0.462 ± 0.016)	0.402–0.548 (0.478 ± 0.041)	0.466–0.592 (0.500 ± 0.047)	0.429
FL/SVL	males	0.471–0.535 (0.491 ± 0.024)	0.473–0.564 (0.527 ± 0.031)	0.433–0.535 (0.476 ± 0.037)	0.374–0.418 (0.410 ± 0.027)
	female	0.432–0.488 (0.468 ± 0.021)	0.439–0.567 (0.482 ± 0.045)	0.407–0.491 (0.449 ± 0.033)	0.388
HL/SVL	males	0.341–0.383 (0.359 ± 0.015)	0.313–0.377 (0.341 ± 0.024)	0.268–0.378 (0.330 ± 0.031)	0.226–0.257 (0.240 ± 0.015)
	female	0.311–0.383 (0.344 ± 0.022)	0.272–0.347 (0.304 ± 0.028)	0.272–0.304 (0.289 ± 0.011)	0.236
HW/SVL	males	0.336–0.401 (0.357 ± 0.023)	0.335–0.394 (0.364 ± 0.021)	0.286–0.369 (0.338 ± 0.029)	0.300–0.351 (0.331 ± 0.023)
	female	0.338–0.388 (0.351 ± 0.017)	0.295–0.360 (0.336 ± 0.019)	0.283–0.354 (0.312 ± 0.025)	0.308
HL/HW	males	0.909–1.077 (1.011 ± 0.063)	0.855–1.030 (0.937 ± 0.058)	0.897–1.191 (0.980 ± 0.092)	0.685–0.758 (0.724 ± 0.029)
	female	0.920–1.020 (0.980 ± 0.031)	0.779–1.057 (0.907 ± 0.098)	0.808–1.043 (0.929 ± 0.093)	0.765
IOD/SVL	males	0.071–0.091 (0.086 ± 0.007)	0.093–0.135 (0.112 ± 0.015)	0.090–0.132 (0.107 ± 0.013)	0.041–0.049 (0.046 ± 0.003)
	female	0.073–0.084 (0.077 ± 0.003)	0.084–0.137 (0.106 ± 0.018)	0.077–0.163 (0.104 ± 0.032)	0.04
TYD/SVL	males	0.042–0.068 (0.052 ± 0.010)	0.055–0.099 (0.082 ± 0.014)	0.029–0.066 (0.050 ± 0.012)	0.040–0053 (0.049 ± 0.004)
	female	0.035–0.052 (0.043 ± 0.006)	0.062–0.102 (0.080 ± 0.012)	0.034–0.064 (0.051 ± 0.010)	0.04
EYD/SVL	males	0.109–0.138 (0.122 ± 0.010)	0.091–0.143 (0.116 ± 0.017)	0.094–0.140 (0.112 ± 0.015)	0.095–0.121 (0.108 ± 0.010)
	female	0.095–0.119 (0.104 ± 0.008)	0.082–0.118 (0.100 ± 0.012)	0.077–0.132 (0.096 ± 0.019)	0.097

**Comparisons.** The following set of morphological characters clearly separates adult *Phrynoglossus swanbornorum* sp. nov. from all congeners: the presence of a rounded canthus rostralis, short snout (SL/SVL ratio 8.4%), rounded tongue without notch, small and indistinct tympanum, granularly textured dorsum with scattered tubercles, dorsal tubercles on flanks tipped in brownish white to white, and the absence of a lateral line, finger discs, tarsal tubercle, inner and outer palmar tubercles, fringe of skin on outer side of fifth metatarsal, and tarsal fold.

When compared against the published morphological characteristics of all other described species of *Phrynoglossus* (also compiled in Appendix), the new species is most morphologically similar to the *P. martensii* complex and differs from its congeners as follows (condition for *P. swanbornorum* sp. nov. in parentheses):

*Phrynoglossus baluensis* exhibits an inverted U-shaped ridge on the dorsum (absent), has paired vocal sacs (singular), relative finger lengths of I ≤II (II<1), has an outer metatarsal tubercle (absent), and its venter is rugose with brown blotches (smooth, uniform cream white).

*Occidozyga berbeza* is a smaller frog reaching 16–18 mm SVL in males and 16–19 mm SVL in females (males 23.2–28.6 mm; females 30.6 mm), exhibits a single tooth-like projection at the tip of the mandible (absent), has relative finger lengths of II<I<IV<III (IV<II<I<III), has pear shaped toe discs (absent), has a fringe on skin of metatarsals (absent), and exhibits a tarsal fold (absent).

*Phrynoglossus celebensis* lacks a canthus rostralis (rounded), has nostrils equidistant between eyes and tip of snout (closer to tip of snout than eyes), has vertically oriented eyes (laterally), has finger discs (absent), and fully webbed toes (moderately webbed).

*Phrynoglossus diminutivus* is a smaller frog reaching 18.6 mm SVL in males and 26.4 mm SVL in females (males 23.2–28.6 mm; females 30.6 mm), exhibits a single tooth-like projection at the tip of the mandible (absent), has paired vocal sacks (singular), and has a fringe on skin of metatarsals (absent).

*Phrynoglossus floresianus* is a larger frog reaching 52.6 mm SVL (males 23.2–28.6 mm; females 30.6 mm), lacks a canthus rostralis (rounded), has nostrils equidistant between eyes and tip of snout (closer to tip of snout than eyes), exhibits fingertips with large discs (absent), has relative finger lengths of II<I<IV (IV<II<I), has fully webbed toes (moderate), and exhibits large toe discs (absent).

*Phrynoglossus laevis* is a larger frog reaching 21–37.4 mm SVL in males and 31.6–48 mm SVL in females (males 23.2–28.6 mm; females 30.6 mm), lacks a canthus rostralis (rounded), has vertically oriented eyes (laterally), has finger discs (absent), two palmar tubercles (one), toe tips with disc (absent), a fringe on skin of metatarsals (absent), a tarsal fold (absent), and retains a lateral line (absent).

*Occidozyga lima* is a larger frog reaching 21–37.4 mm SVL in males and 31.6-48 mm SVL in females (males 23.2–28.6 mm; females 30.6 mm), lacks a canthus rostralis (rounded), exhibits a single tooth-like projection at the tip of the mandible (absent), has a tongue that is pointed behind (rounded), vertically oriented eyes (laterally), nostrils that are equidistant between eyes and tip of snout (closer to tip of snout than eyes), an indistinct supratympanic fold (distinct), lacks distal subarticular tubercles on fingers III and IV (present), has toe tips with discs (absent), an outer metatarsal and a tarsal tubercle (absent), a tarsal fold (absent), retains lateral line system (absent), and has a venter covered in pearly tubercles (smooth).

*Phrynoglossus magnapustulosus* is a smaller frog reaching 17–20 mm SVL in males and 14–21.3 mm SVL in females (males 23.2–28.6 mm; females 30.6 mm), lacks a canthus rostralis (rounded), has two inner palmar tubercles (single), a fringe on skin of metatarsals (absent), and a tarsal fold (absent).

*Phrynoglossus martensii* females reach up to 45 mm SVL (30.6 mm), lacks a canthus rostralis (rounded), has an interorbital distance wider than the internarial distance (interorbital region smaller than upper eyelid), an interorbital distance about three times wider than upper eyelid (interorbital region smaller than internarial distance), has relative finger lengths of II =IV (IV<II), distal subarticular tubercles on fingers III and IV (absent), relative toe lengths of I<II<III<V<IV (I<V<II<III<IV), a fringe of skin on outer side of fifth metatarsal (absent), and a tarsal fold (absent).

*Phrynoglossus myanhessei* has an interorbital distance greater than width of upper eyelid (interorbital distance smaller than upper eyelid), internasal distance less than interorbital distance (internasal distance greater than interorbital distance), relative finger lengths of II<IV<I<III (IV<II<I<III), palmar tubercles are bifid (singular), relative toe lengths of I<II<V<II<IV (I<V<II<III<IV), has toes that are almost fully webbed (moderately webbed), an outer metatarsal tubercle (absent), a fringe on skin of metatarsals (absent), a tarsal fold (absent), and the dorsolateral stripe is absent (sometimes present).

*Phrynoglossus semipalmatus* females reach up to 35–48 mm SVL (30.6 mm), lacks a canthus rostralis (rounded), exhibits a single tooth-like projection at the tip of the mandible (absent), has nostrils equidistant between eyes and tip of snout (closer to tip of snout than eyes), fingertips with discs (absent), relative finger lengths I<II =IV (IV<II<I<III), toe tips with discs (absent), feeble fringe of skin on metatarsals (absent), feeble tarsal fold (absent).

*Phrynoglossus sumatranus* females reach 35–61.6 mm SVL (30.6 mm), exhibits a single tooth-like projection at the tip of the mandible (absent), has vertically oriented eyes, (laterally), an interorbital region smaller than width of the eyelid (interorbital region smaller than the width of upper eyelid), an indistinct supratympanic fold (distinct), relative finger lengths of III<IV (IV<III), toes that are fully webbed to discs (moderately webbed), toe tips with discs (absent), a fringe of skin on metatarsals (absent), retains a weak lateral line system (absent), has a blackish gular (brown with white mottling), and a dark brown band on either side of the cloaca (absent).

*Phrynoglossus tompotika* has a snout equal to diameter of eye (snout smaller than diameter of eye), a loreal region that is concave (convex), exhibits a single tooth-like projection at the tip of the mandible (absent), paired vocal sac (singular), fingers with discs (absent), relative finger lengths of II<I<IV<III (IV<II<I<III), two inner palmar tubercles (singular), relative toe lengths of I<II<V<III<IV (I<V<II<III<IV), toes with discs (absent), toe discs with circummarginal groove (absent), and a tarsal fold (absent).

**Figure 7:**
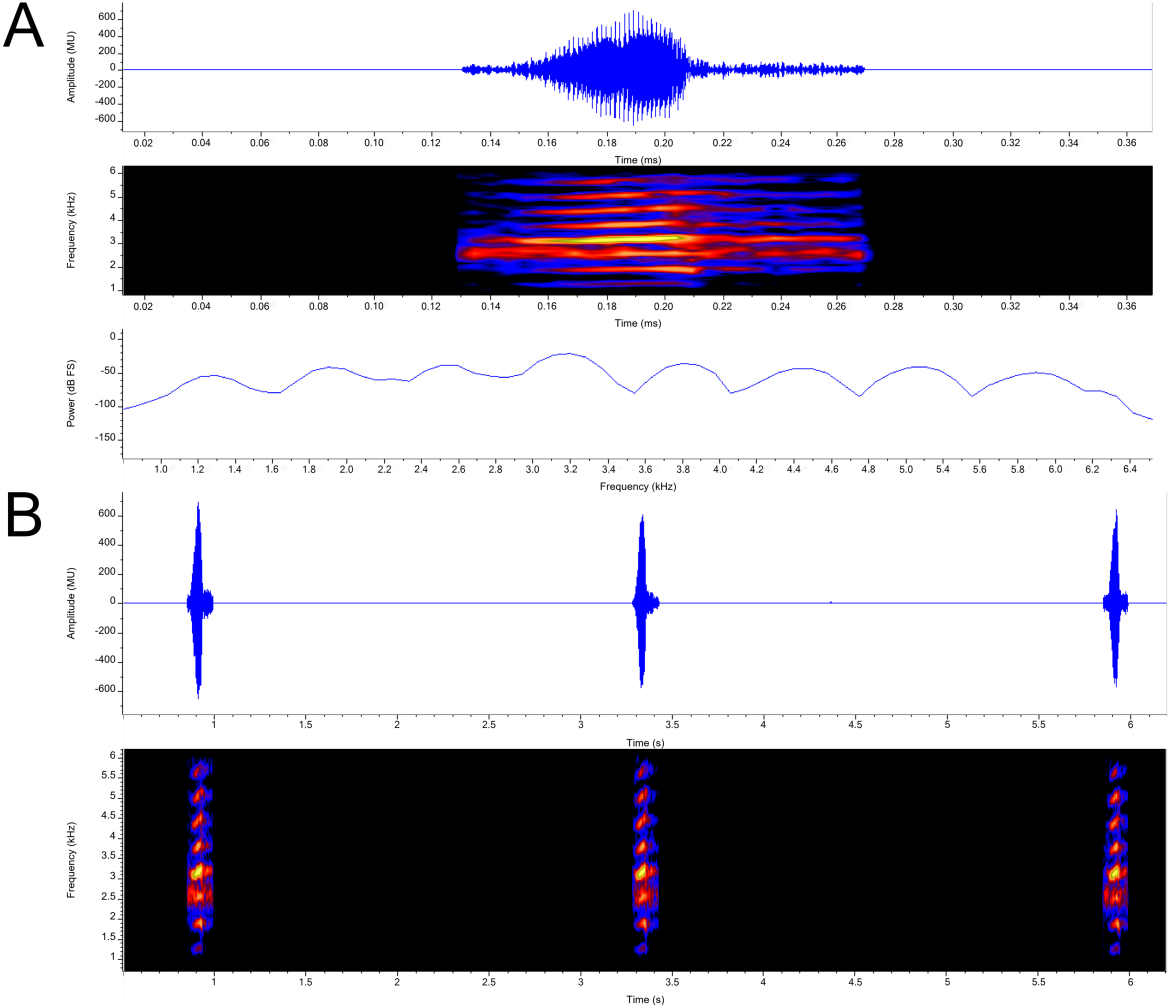
Advertisement calls from a single male *P. swanbornorum* sp. nov. (A) Audio spectrogram, oscillogram, and power spectrum views of a single note (B) audio spectrogram and oscillogram views of multiple notes. Macaulay Library catalogue number ML237498.

**Call (Fig. 7).** Each call is a high-pitched “yip” that consists of single pulsatile note. Call duration measured 0.102 –0.125 s ($\bar {x}$ = 0.114 ± 0.009; *n* = 7) with an intercall duration of 2.315–3.064 s ($\bar {x}$ = 2.628 ± 0.264; *n* = 6). Call period measured 2.426−3.168 s ($\bar {x}$ =2.741 ±0.264; *n* = 6). Call repetition rate measured 0.316 –0.412/s ($\bar {x}$ = 0.365 ± 0.034; *n* = 6). The calls exhibit narrowly separated parallel frequency bands. Calls contain a loosely defined central pulse group (28–31 pulses per call; $\bar {x}$ = 30.143 ± 1.574; *n* = 7) with indistinguishable amplitude modulation present on either end of the call. Pulse group duration measured 0.045–0.052 s ($\bar {x}$ = 0.050 ±0.003; *n* = 7). Pulse rate varied from 593.870 − 622.490/*s* ($\bar {x}$ = 608.947 ± 11.716; *n* = 7). Apart from the pulsatile nature of calls, amplitude modulation is detected, with energy increasing and an occasional (*n* = 5 out of 7) slight drop of energy near the middle of the call, subsequently reaching its maximum energy within 3-5 pulses, then decreasing again. Minimum and maximum frequencies were measured at 5% and 95% respectively and ranged from 2,498-4,393 Hz with both maximum call energy and maximum frequency at 3,187 Hz; *n* = 7.

Call data of congeners have been completely described for *P. sumatranus* (Peters, 1877; Márquez & Eekhout, 2006), *P. magnapustulosus* (Taylor & Elbel, 1958);(Köhler et al., 2021), *P. martensii* populations in Myanmar, and *P. myanhessei* (Köhler et al., 2021). The call of *Phrynoglossus swanbornorum* sp. nov. was recorded at a higher ambient temperature (30.1 C) than that of *P. sumatranus* (24.5–29 C). The call descriptions for *P. magnapustulosus, P. martensii*, and *P. myanhessei* lacked data on climatic condition. The call of *O. lima* (Deesrisai et al., 2015) and of two disjunct populations of the *P. martensii* complex (Wang et al., 2016; Deesrisai et al., 2015) have been partially described but lack terminology definitions, graphic representations, and data on climatic condition. The described calls of *P. martensii* are from three distinct populations: population (1) Huangzhu Town, Ding’an County, Hainan Province, China (Wang et al., 2016); population (2) Khao Ang Rue Nai Wildlife Sanctuary, Chachoengsao Province, Thailand; and population (3) various sites across Myanmar. The call of *P. baluensis* (Boulenger, 1896) is briefly described as a series of low-pitch, raspy notes (Haas, Das & Hertwig, 2020). To compare the call of *O. lima* to *Phrynoglossus swanbornorum* sp. nov., “element” (Deesrisai et al., 2015) is equated to “call” as *O. lima* has a multiple note or “element” call, whereas all other species compared have a single note call.

The call of *Phrynoglossus swanbornorum* sp. nov. is differentiated mainly by having a higher pulse rate ($\bar {x}$ = 608.9/s in *Phrynoglossus swanbornorum* sp. nov.; $\bar {x}$ = 143.2/s in *P. sumatranus*); intermediate intercall duration ($\bar {x}$ = 2.628 s in *Phrynoglossus swanbornorum* sp. nov., $\bar {x}$ = 9.293 s in *P. sumatranus*; $\bar {x}$ = 2.050 s in *P. martensii* population 1; $\bar {x}$ = 3.278 s in *P. martensii* population 2; $\bar {x}$ = 2.77−3.92 s for three individuals of *P. magnapustulosus*; $\bar {x}$ = 0.213 s in *O. lima*); an intermediate call duration ($\bar {x}$ = 0.114 s in *Phrynoglossus swanbornorum* sp. nov.; $\bar {x}$ = 0.052 s in *P. martensii* population 1; $\bar {x}$ = 0.023 s in *P. martensii* population 2; $\bar {x}$ = 0.037−0.046 s for five individuals of *P. martensii* population 3; $\bar {x}$ = 0.090−0.104 s for three individuals of *P. myanhessei*; $\bar {x}$ = 0.277−0.387 s for three individuals of *P. magnapustulosus*; $\bar {x}$ = 0.165 s in *P. sumatranus*; $\bar {x}$ = 0.05 s in *O. lima*); an intermediate dominant frequency (3,187 Hz in *Phrynoglossus swanbornorum* sp. nov.; 3,782 Hz in *P. martensii* population 1; 3,444–3,914 Hz for five individuals of *P. martensii* population 3; 2,467–2,839 Hz for three individuals of *P. myanhessei*; 3,447–3,761 Hz for three individuals of *P. magnapustulosus*; 2,742 Hz in *P. sumatranus*); higher minimum frequency (2,498 Hz in *Phrynoglossus swanbornorum*; 1,708–1,894 Hz in three individuals of *P. magnapustulosus*; 1,529–2,119 in three individuals of *P. myanhessei*); lower maximum frequency (3,187 Hz in *Phrynoglossus swanbornorum* sp. nov.; 11,419 Hz in *P. martensii* population 2; 3,830–4,493 Hz for five individuals of *P. martensii* population 3; 4,150–4,823 Hz for three individuals of *P. magnapustulosus*; 2,839–3,265 Hz for three individuals of *P. myanhessei*; 3,470 Hz in *O. lima*); and single note call in *Phrynoglossus swanbornorum* sp. nov., *P. sumatranus*, and *P. martensii*, whereas *O. lima* has a multiple note call.

**Etymology.** The specific patronym epithet “swanbornorum” is in genitive plural and refers to members of the Swanborn family, who are generous supporters of the conservation efforts of the Creative Conservation Alliance.

**Distribution.***Phrynoglossus swanbornorum* sp. nov. is currently only confirmed to occur within the Chunati Wildlife Sanctuary (21.937533 N, 92.063010 E), Chattogram District at an elevation of ca. 33 m and Teknaf Wildlife Sanctuary (20.887020 N, 92.298954 E), Cox’s Bazar District at an elevation of ca. 26 m (Fig. 1).

### Natural history

*Phrynoglossus swanbornorum* sp. nov. is only known to inhabit lowland, coastal, mixed-evergreen forests of southeast Bangladesh, which are bounded to the east by the mountainous Chattogram Hill Tracts. Within this habitat, *Phrynoglossus swanbornorum* sp. nov. has only been observed near lentic water bodies under dense forest canopy and was notably absent from surrounding agricultural fields and degraded forest. An image of *P. swanbornorum* sp. nov. in amplexus (see Distribution; Ahm, 2014) demonstrates that this species exhibits inguinal amplexus. Additionally, eggs from the single collected female were pigmented.

## Discussion

### Phylogeny

*Phrynoglossus swanbornorum* sp. nov. formed a distinct clade from neighbouring congeners *P. martensii*, *P. magnapustulosa*, *P. myanhessei*, and an undescribed species from Yangon, Myanmar. The closest known congener is the undescribed species from Myanmar, last collected in 2001, and sequenced by Mulcahy et al. (2018) which was recognized as a distinct species “*Occidozyga* sp. A” (MG935919, MG935914, MG935921) based on their analysis of the COI gene. Following the nomenclature of Köhler et al., 2021, the *Phrynoglossus* clade formed a monophyly and only two *Occidozyga* species are recognized—*O. lima* and *O. berbeza*. The node supporting the *Occidozyga* monophyly is weak with Bayesian posterior probabilities (BPP < 0.80) and the bootstrap supports (BS < 70). The addition of nuclear genetic data would likely assist in evaluation of the relationship between *Phrynoglossus* and *Occidozyga*.

### Morphology

Morphological descriptions of *Phrynoglossus* spp. and *Occidozyga* spp. in the peer reviewed literature were found to be incomplete and, in some cases, incongruent. We provide a compilation of morphological characters (see Appendix) from the peer reviewed literature, supplemented by grey literature, which allows for the modification of the generic diagnosis by elucidating morphological synapomorphies.

Where character state is reported, all *Phrynoglossus* and *Occidozyga* species are found to lack vomerine teeth and have nuptial pads/spines, a bony style, a forked omosternum, pigmented eggs, a distinct or indistinct supratympanic fold, reduced to absent metacarpal webbing, moderate to extensive metatarsal webbing, elongated inner metatarsal tubercle, short arms, and small tympanum covered with skin. Most species also lack a distinct canthus rostralis with the exceptions of *Phrynoglossus swanbornorum* sp. nov. and *P. tompotika* (Iskandar, Arifin & Rachmansah, 2011). Only four species are reported to have finger discs, while toes discs are present, at least feebly, in all species. Additionally, both paired and single vocal sacs have been reported and Köhler et al. (2021) describes *Phrynoglossus* as having an extensive mucosome whereas *O. lima* has a diminished mucosome. Lastly, *Phrynoglossus* species have been found to reach between 15 and 61.6 mm in length with females being larger than males and *P. sumatranus* reaches the greatest length.

Considering these findings, multiple characters used in previous diagnoses of *Phrynoglossus* and *Occidozyga* (Taylor, 1962; Iskandar, 1998; Sailo et al., 2009; Inger et al., 2017; Poyarkov et al., 2020) are found to be insufficiently descriptive and should no longer be used. For instance, the previously used character of the length of finger I being equal or subequal to finger II applies only to *P. diminutivus* (Taylor, 1922), *O. berbeza*, and *O. lima*. Likewise, not all species exhibit a tuberculate dorsum, *i.e., P. diminutivus* exhibits a smooth dorsum and tuberculate flanks. Additionally, not all species exhibit a smooth ventrum as *P. sumatranus* has been reported to have a rugose ventrum (Inger, 1966). Lastly, not all species lack circummarginal grooves; *P. tompotika* exhibits circummarginal grooves on toe disks.

The pupils of several species were commonly described as either ovoid or diamond shaped, and both Iskandar, Arifin & Rachmansah (2011) and Köhler et al. (2021) describe these character states as diagnostic for *Phrynoglossus*. It is now known that both diamond and ovoid can be varying states of the same character within *Phrynoglossus* as the pupil of an individual *Phrynoglossus swanbornorum* sp. nov. was observed to change from ovoid, when dilated, to diamond shaped when constricted (Figs. 5A, 5B). Therefore, it is likely that these species exhibit horizontal pupils which resemble a diamond shape when constricted and ovoid when dilated.

Several *Phrynoglossus* species exhibit the paedomorphic trait of lateral line retention, though it has been previously misreported in the literature and its state is not reported for seven *Phrynoglossus* species. Dubois, Ohler & Biju (2001) correctly claimed that the lateral line system is retained in *Occidozyga* (*sensu* Dubois) and is absent in *Phrynoglossus* (*sensu* Dubois), and a review of the literature confirms this (see Appendix). Frost et al. (2006) incorrectly cited Dubois, Ohler & Biju (2001) stating that the lateral line system was a synapomorphy for Occidozyginae and was retained in *Phrynoglossus* (*sensu* Dubois) and absent in *Occidozyga* (*sensu* Dubois). Additionally, in species where it has been described, the presence of a lateral line system corresponds with the presence of dorsally oriented eyes and nares, whereas its absence corresponds with laterally oriented eyes and nares. Therefore we predict a lateral line system to be present in *P. celebensis* (Smith, 1927) and absent in *O. berbeza*, *P. baluensis*, *P. diminutivus*, *P. floresianus* (Mertens, 1927), *P. magnapustulosus*, *P. myanhessei*, *P. semipalmatus* (Smith, 1927), and *P. tompotika.*

Detailed descriptions of several other characters are also lacking in the literature for *Phrynoglossus* and *Occidozyga*. The presence or absence of several types of tubercle have not been reported for nearly half the species including nuptial pads, subarticular tubercles, tarsal tubercle, and palmar tubercles. Other characters not reported for nearly half these species include color of egg, fringe of skin on metatarsals, interorbital distance, internasal distance, pupil shape, and whether a single tooth-like process/projection is present at the tip of the mandible. Some characters are reported, yet lack sufficient clarification to compare e.g., subarticular tubercles are rarely defined as those found on the toes or the fingers and vocal sac descriptions lack specification of whether they are internal or external.

A number of incongruent descriptions were discovered in the literature and from unpublished photographs (see Appendix). *Phrynoglossus baluensis* was reported as lacking a canthus rostralis (Boulenger, 1896) yet also having a rounded canthus rostralis (Inger, 1966). *Phrynoglossus celebensis* is reported to (1) have a tongue that is not rounded (Iskandar, Arifin & Rachmansah, 2011), yet also a tongue that is rounded or feebly nicked behind (Smith, 1927; Smith, 1931) (2) fingertips with discs (Smith, 1931), yet also fingertips with conical tips (Matsui et al., 2021). *Phrynoglossus floresianus* is described as having a tongue that is notched behind (Iskandar, Arifin & Rachmansah, 2011), yet also rounded or feebly nicked behind (Smith, 1931; Mertens, 1927). *Phrynoglossus laevis* is described as having (1) toe tips with no disks (Iskandar, Arifin & Rachmansah, 2011), yet also toe tips with distinct discs (Bourret, 1942; Inger, 1954; Taylor, 1962); (2) toes rather elongate (Taylor, 1962), yet also short toes (Alcala & Brown, 1998); and (3) relative finger lengths as II<=I (Bourret, 1942; Inger, 1954), yet also I<II (Taylor, 1962). *Phrynoglossus martensii* was described as (1) having a head wider than its length (Taylor, 1958) yet also longer than its width (Hecht et al., 2013); (2) a supratympanic fold that is sharp and distinct (Gawor et al., 2016) yet also weakly developed (Hecht et al., 2013); and (3) tarsal fold distinct (Taylor, 1962) yet also absent (Andersson, 1942). *Phrynoglossus sumatrana* was reported as (1) lacking a toothlike projection on the tip of the mandible (Iskandar, 1998) yet also possessing one (Inger et al., 2017); (2) having small toe discs (Iskandar, Arifin & Rachmansah, 2011), yet also “distinct enlarged digital disc[s]” (Iskandar, 1998); (3) having smooth ventral skin (Iskandar, 1998), yet also rugose ventral skin (Inger, 1966); and (4) having a broadly rounded and indistinct canthus rostralis (Inger, 1966), yet also lacking one (Peters, 1877). While some discrepancies may be the result of inter-observer error, they might also be indicative of undescribed cryptic diversity or intraspecies variation.

### Amplexus

**Figure 8:**
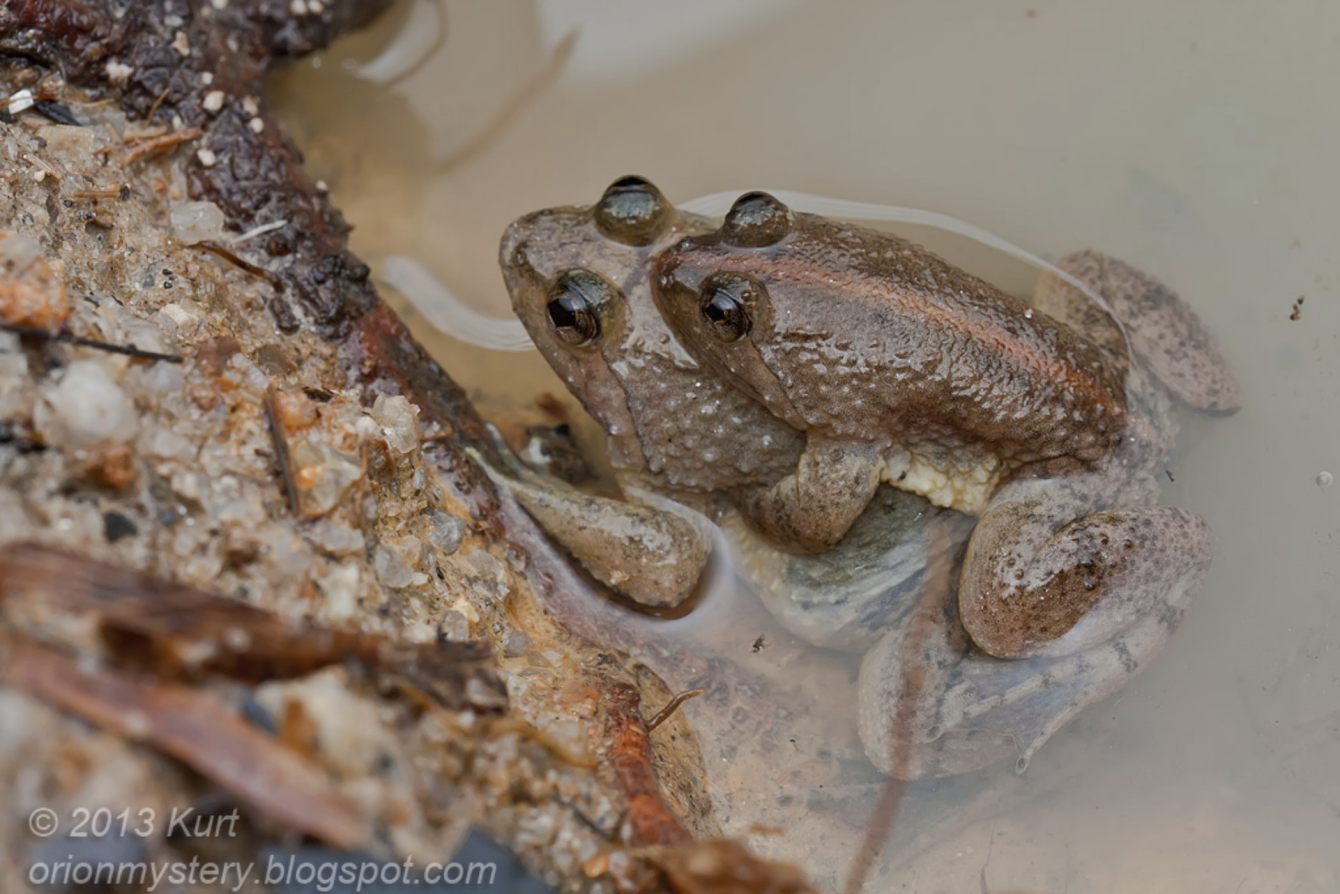
Axillary amplexus in *P. martensii*. Photographed by GUEK Hock Ping (Kurt) in Selangor, Malaysia. Used with permission.

**Figure 9:**
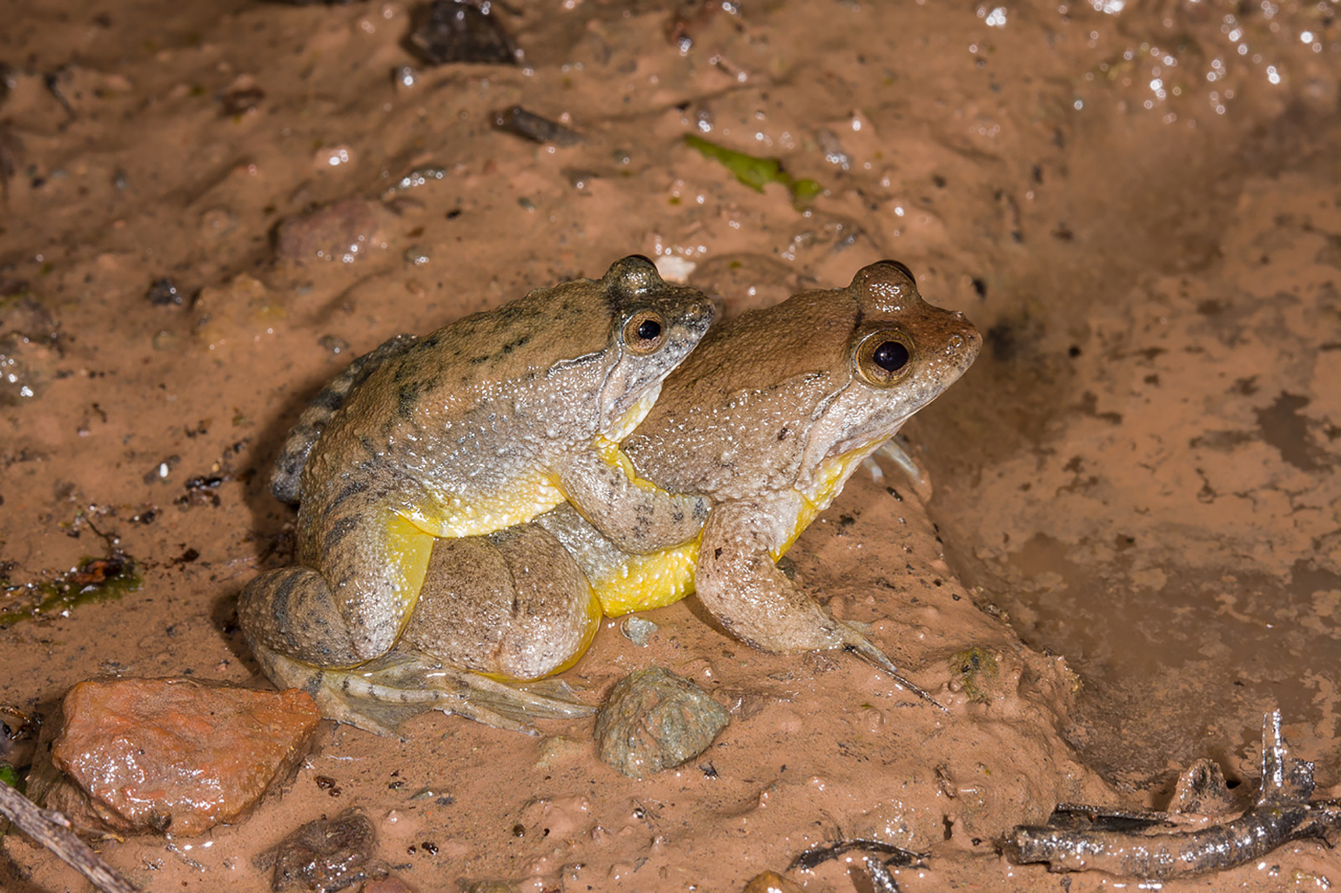
Axillary amplexus in *P. magnapustulosus*. Photographed by Chris Mattison in Borneo. Used with permission.

*Phrynoglossus magnapustulosus* and *P. myanhessei* were recently described as exhibiting inguinal amplexus (Köhler et al., 2021). Coupled with the observation of inguinal amplexus in *P. sumatranus* (Eto & Matsui, 2012), this lead Köhler et al. (2021) to claim inguinal amplexus as an autapomorphy for *Phrynoglossus*. Herein, we present evidence to the contrary with observations of axillary amplexus in *P. martensii* (Fig. 8) and *P. sumatrana* (Fig. 9)*.* Therefore, we assert that inguinal amplexus is not an autapomorphy for *Phrynoglossus*, though it may be a useful secondary sexual character for diagnosing cryptic species.

In addition to axillary amplexus, *P. martensii* has also been reported to exhibit both inguinal amplexus (Ziegler, 2000; Chan-Ard, 2003) and lumbar amplexus (Sailo et al., 2009), though we believe that the usage of the term “lumbar amplexus” here may be analogous to inguinal amplexus as Blommers-Schlösser (1981) has similarly also equated the two terms and true lumbar amplexus is a rare and primitive trait (Laurent, 1964).

### Distribution

No *Phrynoglossus* species has been previously reported in South Asia, and the only *Occidozyga* species reported in South Asia is *O. lima*. Several publications vaguely refer to *Occidozyga* occurring in South Asia, specifically “Bengal,” which historically refers to much of eastern India, but contemporary usage of “Bengal” would be restricted to the state of West Bengal, India or in Bangladesh. According to Günther (1864) *Oxyglossus* [*Occidozyga* ] *lima* is “said to occur also in Bengal,” which was restated by Boulenger (1890) in his description of *O. lima* as inhabiting “Lower Bengal.” Sarkar, Biswas & Ray (1992) cites Boulenger (1890) and restates that *O. lima* occurs in “West Bengal” with no additional observations reported.

Khan (2001) describes *O. lima* as commonly encountered, yet our survey efforts in the Teknaf peninsula yielded only *Phrynoglossus swanbornorum* sp. nov*. Occidozyga lima* is also reported from the Chattogram Hill Tracts in southeast Bangladesh within Kaptai National Park (Ahm, 2014; Reza & Perry, 2015). Photographic evidence is provided in Ahm (2014) of two individuals, however the individuals photographed are unequivocally not *O. lima* due to their laterally oriented eyes. Despite being unable to definitively identify these individuals to species level solely based on these photographs, they exhibit characteristics congruent with those of *P. swanbornorum* sp. nov. and are reported from within the predicted range of *P. swanbornorum* sp. nov. Therefore, we suspect that all reports of *O*. *lima* from southeast Bangladesh (Khan, 1997; Khan, 2001; Ahm, 2014; Khan, 2016; Reza & Perry, 2015) are instead *P. swanbornorum* sp. nov.

*Occidozyga lima* was also reported from northern Bangladesh in Nilphamari District (Kabir, Hawkeswood & Makhan, 2020), though all photographs of these observations were lost, and no specimens were taken (A. Kabir, 2020, pers. comm.). However, extensive herpetofaunal sampling efforts in northeast Bangladesh did not yield observations of *O. lima* (Hakim et al., 2020). Likewise, informal surveys conducted in central Bangladesh within Bhawal National Park and in northwest Bangladesh within rural areas near Rajshahi did not detect the presence of *Occidozyga* or *Phrynoglossus* (S Trageser, S Rahman, 2011-2019, SCR personal observations). Furthermore, surveys described in Khan (2001) did not observe these genera within the Sylhet region in northeast Bangladesh. No observations of *Occidozyga* are known between Nilphamari District and the northernmost known locality of Kaptai National Park, a distance of approximately 480 km, despite regions between these two observations having been surveyed. The observations reported by Kabir, Hawkeswood & Makhan (2020) are likely that of either an introduced population or are a misidentification of another taxon. Thus, no individuals of *Occidozyga lima* or *Phrynoglossus* spp. have been reported in Bangladesh outside of the Chattogram Division, despite surveys in the moist, deciduous sal (*Shorea robusta*) forest of central Bangladesh, the Sundarbans mangrove system in south-central Bangladesh, and the semi-evergreen hill forests of northeast Bangladesh (S Trageser, S Rahman, 2011-2019, SCR personal observations). Therefore, the range of the genera *Occidozyga* and *Phrynoglossus* likely does not extend to West Bengal.

We have confirmed the occurrence of *P. swanbornorum* in both Chunati and Teknaf Wildlife Sanctuaries and the aforementioned probable records from Kaptai National Park, 55 km to the north of Chunati Wildlife Sanctuary, would represent the furthest observation to the northwest of any *Phrynoglossus* spp. Additionally, *Phrynoglossus swanbornorum* sp. nov. appears to be the westernmost distributed species of *Phrynoglossus*, so this genus presumably reaches its western limit in the lowland forests of southeast Bangladesh. Considering the biogeography of the region, such a distribution is congruent with the hypothesis that the lower Ganges and Brahmaputra River act as barriers to dispersal, thereby separating many faunal lineages of South and Southeast Asian decent (Reza, 2010; Trageser et al., 2017).

### Conservation and threats

We suggest this taxon be listed within Bangladesh as EN based on criteria B1ab(i,ii,iii,iv) + 2ab(i,ii,iii,iv), as it has an estimated area of occupancy (AOO) of 32 km^2^ and extent of occurrence (EOO) of 31,284 km^2^. The provided AOO and EOO include confirmed locations of Chunati and Teknaf Wildlife Sanctuaries, historical points from Myanmar, and the probable locality of Kaptai National Park. The status would not change if the assessment did not include the Myanmar specimens. Additionally, the number of subpopulations is inferred to be experiencing continuing decline due to ongoing habitat destruction, agricultural contamination, vehicular mortalities, mortality *via* frequent foot traffic in breeding pools within walking trails, and depredation by domestic dogs. Similar threats are known to impact *P. floresianus* and *P. tompotika* populations, which are considered Vulnerable (IUCN SSC Amphibian Specialist Group, 2019a; IUCN SSC Amphibian Specialist Group, 2019b) and Critically Endangered (IUCN SSC Amphibian Specialist Group, 2019a; IUCN SSC Amphibian Specialist Group, 2019b), respectively. Chunati and Teknaf Wildlife Sanctuaries and Kaptai National Park offer formal protection for *Phrynoglossus swanbornorum* sp. nov., unfortunately, these sanctuaries currently lack enforcement to adequately protect *Phrynoglossus swanbornorum* sp. nov. habitat. Satellite imagery analysis indicates that there are no intact forested landscapes within the known range of *Phrynoglossus swanbornorum* sp. nov.*,* and sufficiently dense forested habitat is severely fragmented, including within these protected areas (Global Forest Watch, 2014). Lowland coastal forests are imperiled habitats in Bangladesh (Global Forest Watch, 2014) and the predicted rise in temperature and precipitation variability due to climate change will likely exacerbate their plight, further reducing available habitat (Shishira et al., 2020).

## Conclusions

We provide morphological, molecular, and bioacoustic evidence that support the validity of the new species, *Phrynoglossus swanbornorum* sp. nov. This species is currently only confirmed from a relatively small area in southeastern Bangladesh and is considered to have a high risk of extinction due to conspicuous and ongoing threats to the habitat it relies on. We also review and discuss inconsistencies in the literature regarding the genera *Phrynoglossus* and *Occidozyga*, particularly that of morphological characters and provide more accurate generic diagnoses for both.

Although multiple species of *Phrynoglossus* and *Occidozyga* are generally perceived to be widespread and common, this study adds to a growing body of literature demonstrating the existence of undescribed diversity within the genus, thereby masking the true extinction risk of these species (Mulcahy et al., 2018). We suggest further research be conducted to either rectify incongruent descriptions of diagnostic characters and/or better understand intraspecific variation within the genus as well as including additional nuclear genetic data. Doing so could result in further refinement of the provided generic diagnoses. We also suggest that further surveys be conducted in the northern part of Chattogram Hill Tracts, Bangladesh e.g., Pablakhali Wildlife Sanctuary and Kassalong Reserve Forest, and throughout Rakhine State, Myanmar to better understand the distribution of *Phrynoglossus swanbornorum* sp. nov.

##  Supplemental Information

10.7717/peerj.11998/supp-1 Supplemental Information 1 Occidozyga swanbornorum sequences

10.7717/peerj.11998/supp-2 Supplemental Information 2 Compilation of morphological characters for all 14 species of *Occidozyga and Phrynoglossus* Character states were sourced from peer reviewed literature and supplemented by grey literature.

## Additional Information and Declarations

### Field Study Permissions

The following information was supplied relating to field study approvals (i.e., approving body and any reference numbers): Field experiments were approved by the Bangladesh Forest Department (22.01.0000.101.23.2019.1922).

### Data Availability

The following information was supplied regarding data availability: The 16S rRNA sequences are available at GenBank: MN075433, MN075434, MN075435, MN075436, and MN075437. Specimens are stored at the Department of Zoology of Jagannath University with the following accession numbers: JnUZool-A0719, JnUZool-A0819, JnUZool-A0919, JnUZool-A1019, and JnUZool-A1117.

### New Species Registration

The following information was supplied regarding the registration of a newly described species: Publication LSID: urn:lsid:zoobank.org:pub:DE8850C6-0A7B-4EA2-AC9E-69EE6EFBF754 Phrynoglossus swanbornorum sp. nov. LSID: urn:lsid:zoobank.org:act:B54285D5-E598-441F-818F-169C2ECF7F99.
